# Supplementary material for: Planimetric and Volumetric Brainstem MRI Markers in Progressive Supranuclear Palsy, Multiple System Atrophy, and Corticobasal Syndrome. A Systematic Review and Meta-Analysis
Source: Neurol Int. 2023 Dec 19;16(1):1–19. doi: 10.3390/neurolint16010001 (PMC10892270; doi:10.3390/neurolint16010001)
Supplement: Supplementary file 1 [file neurolint-16-00001-s001.zip › neurolint-2742391-supplementary.pdf]

Supplementary Table 1. Analytical presentation of study characteristics regarding the signaling questions, risk of bias and concerns regarding applicability for patient selection, Index Test, Reference Standard and Flow/Timing, based on the QUADAS Tool; ROB: Risk of Bias; App: Concerns regarding applicability; : low; : high; ?: unclear.

		Patient Selection				Index Test			Reference Standard				Flow and Timing			
		Consecutive / random sample	Innapropriate exclusions	ROB	App	Test assessed blindly	ROB	App	RS classify participants	RS assessed blindly	ROB	App	All patients RS	All same RS	All patients included	ROB
1	Oba, 2005	?	☺	?	☺	?	?	☺	☺	?	☺	☺	☺	☺	☺	☺
2	Groschel, 2006	☺	☺	☺	☺	?	☺	☺	☺	?	☺	☺	☺	☺	☺	☺
3	Paviour, 2006	?	☺	?	☺	?	?	☺	☺	?	☺	☺	☺	☺	☺	☺
4	Cosottini, 2007	?	☺	?	☺	?	?	☺	☺	?	☺	☺	☺	☺	☺	☺
5	Borroni, 2010	☹	☹	☹	☺	?	?	☺	☺	?	☺	☺	☺	☺	☺	☺
6	Longoni, 2010	?	☺	?	☺	☺	☺	☺	☺	☺	☺	☺	☺	☺	☺	☺
7	Looi, 2011	?	☺	?	☺	☺	☺	☺	☺	?	☺	☺	☺	☺	?	☺
8	Morelli , 2011	?	☺	?	☺	☺	☺	☺	☺	?	☺	☺	☺	☺	☺	☺
9	Morelli, 2014	?	☺	?	☺	☺	☺	☺	☺	?	☺	☺	☺	☺	?	☺
10	Huppertz, 2016	?	☺	?	☺	?	?	☺	☺	?	☺	☺	☺	☺	☺	☺
11	Magnesius, 2016	?	☺	?	☺	☺	☺	☺	☺	?	☺	☺	☺	☺	?	☺
12	Pasha, 2016	☺	☹	☹	☺	?	?	☺	☺	?	☺	☺	☺	☺	☺	☺
13	Sankhla, 2016	?	☺	?	☺	☺	☺	☺	☺	?	☺	☺	☺	☺	☺	☺
14	Nigro, 2017a	?	☺	?	☺	☺	☺	☺	☺	?	☺	☺	☺	☺	☺	☺
15	Nigro, 2017b	?	☺	?	☺	☺	☺	☺	☺	?	☺	☺	☺	☺	☺	☺
16	Nizamani, 2017	☺	☺	☺	☺	☺	☺	☺	☺	?	☺	☺	☺	☺	☺	☺
17	Sislby, 2017	?	☺	?	☺	☺	☺	☺	☺	?	☺	☺	☺	☺	☺	☺
18	Quattrone, 2018	☺	☺	☺	☺	☺	☺	☺	☺	☺	☺	☺	☺	☺	☺	☺
19	Ahn, 2019	?	☺	?	☺	☺	☺	☺	☺	?	☺	☺	☺	☺	☺	☺
20	Krismer, 2019	?	☺	?	☺	☺	☺	☺	☺	☺	☺	☺	☺	☺	☺	☺
21	Quatrone, 2019	☺	☺	☺	☺	☺	☺	☺	☺	?	☺	☺	☺	☺	☺	☺
22	Constantinides, 2019	☺	☺	☺	☺	?	?	☺	☺	?	☺	☺	☺	☺	☺	☺
23	Jabbari, 2020	?	☺	?	☺	☺	☺	☺	☺	?	☺	☺	☺	☺	☺	☺
24	Nigro, 2020	?	☺	?	☺	☺	☺	☺	☺	?	☺	☺	☺	☺	☺	☺
25	Madetko, 2022	?	☺	?	☺	?	?	☺	☺	?	☺	☺	☺	☺	?	☺

26	**Virahammar, 2022**	?	☺	?	☺	☺	☺	☺	☺	?	☺	☺	☺	☺	☺	☺
27	**Quattrone, 2023**	☺	☺	☺	☺	☺	☺	☺	☺	?	☺	☺	☺	☺	☺	☺

Supplementary Table 2. Analytical data regarding the number of patients per study group, mean values and standard deviations of all available MRI markers in studies of Richardson patients included in the meta-analysis.

Study number	Study ID	Richardson's Syndrome								Controls							
		N	midbrain area	pons area	M/P area	MRPI 1	MRPI 2	midbrain volume	pons volume	N	midbrain area	pons area	M/P area	MRPI 1	MRPI 2	midbrain volume	pons volume
1.	Oba, 2005	21	56 (7.6)	448.6 (52.1)	0.12 (0.02)					31	117.7 (14.7)	570 (42.7)	0.24 (0.03)				
2.	Groschel, 2006	33	81.1 (13.6)							22	138.1 (25.6)						
3.	Paviour, 2006	18						5.7 (1.1)	12.8 (1.6)	18						8.3 (0.8)	13.9 (1.5)
4.	Cosottini, 2007	15	90 (17)	507 (56)	0.18 (0.04)			4.0 (0.7)		14	155 (15)	570 (45)	0.28 (0.04)			5.9 (1.7)	
5.	Borroni, 2010	18			0.19 (0.03)					25			0.255 (0.03)				
6.	Longoni, 2011	10	71 (27)	512 (94)	0.129	20.7 (7.1)				24	130.5 (30.5)	537.8 (61.1)	0.233	10.5 (2.4)			
7.	Looi, 2011	15	93.8 (20.1)	526 (62)	0.178					15	145.3 (20.1)	577.4 (61.6)	0.252				
8.	Morelli, 2011	42	64 (13.9)	417 (57)	0.15 (0.03)	21.4 (7.4)				38	122 (17.3)	473 (47.3)	0.26 (0.04)	9.4 (1.8)			
9.	Morelli, 2014	25	77.3 (14.6)	472 (58)	0.163 (0.02)	18.9 (4.4)				81	142.4 (21.2)	528.8 (51.6)	0.27 (0.04)	9.2 (1.6)			
10.	Huppetz, 2016	106	136.1 (22.5)	450 (59)				8.9 (0.9)	13.9 (1.6)	73	170 (11.3)	511.6 (45.2)				10.5 (0.6)	15.8 (1.4)
11.	Pasha, 2016	17	81.1 (23.6)	499 (59)						30	146.1 (15.6)	504.9 (53.5)					
12.	Sankhla, 2016	26	71.9 (16.5)	461 (61)	0.16 (0.03)	23.5 (9.6)				30	135.6 (21.7)	495.5 (37.7)	0.27 (0.04)	9.5 (1.9)			
13.	Nigro, 2017a (1/2 - 1.5T)	44	74 (15)	465 (53)		18.3 (5.3)				19	127 (19)	500 (43)		10.1 (1.1)			
	Nigro, 2017a (2/2 - 3T)	37	72 (14)	459 (58)		20.6 (5.6)				92	134 (18)	526 (55)		9.5 (1.4)			
14.	Nigro, 2017b	15	62 (13)	465 (70)	0.13 (0.02)	27.0 (5.8)				86	138 (18)	524 (53)	0.26 (0.03)	9.3 (1.5)			
15.	Nizamani, 2017	34	96 (13.5)	431 (49)						34	149.4 (12.5)	457.6 (38.1)					
16.	Silsby, 2017	16	88.3 (22)	486 (52)	0.18 (0.04)	17.6 (4.1)				22	152.6 (16.3)	523.8 (45.2)	0.29 (0.03)	10.3 (2.0)			
17.	Quattrone, 2018	46				20.4 (4.7)	5.2 (1.7)			53				9.1 (1.3)	1.5 (0.4)		
18.	Ahn, 2019	27	87 (15.8)	507 (39)	0.17 (0.04)					27	129 (18)	517 (54)	0.25 (0.02)				
19.	Quattrone, 2019	48	73.9 (16.2)							38	137.5 (16.4)						
20.	Constantinides, 2019	43	90 (23)		0.18 (0.04)	22.5 (9.0)				29	141.5 (25.9)		0.27 (0.02)	12.5 (4.0)			
21.	Jabbari, 2020	25						5.0 (0.5)		35						6.0 (0.5)	
22.	Nigro, 2020	108	70 (16)	465 (54)		20.1 (5.6)				139	128 (20)	497 (49)		9.6 (2.0)			
23.	Madetko, 2022	19	76 (32)	477 (39)	0.16 (0.05)	19.4 (6.9)	5.6 (2.3)			16	121 (23)	496 (73)	0.25 (0.03)	11.2 (1.8)	2.3 (0.6)		
24.	Virhammar, 2021	29	80.7 (6.7)							38	133.6 (33.6)						
25.	Quattrone, 2023	62				20.2 (5.0)	5.1 (1.9)							9.4 (1.3)	1.4 (0.5)		

Supplementary Table 3. Analytical data regarding the number of patients per study group, mean values and standard deviations of all available MRI markers in studies of MSA-P patients included in the meta-analysis.

Study number	Study ID	MSA-P								Controls							
		N	midbrain area	pons area	M/P area	MRPI 1	MRPI 2	midbrain volume	pons volume	N	midbrain area	pons area	M/P area	MRPI 1	MRPI 2	midbrain volume	pons volume
1.	Oba, 2005	25	97.2 (17.0)	381.6 (97.0)	0.27 (0.07)					31	117.7 (14.7)	570 (42.7)	0.24 (0.03)				
2.	Huppetz, 2016	60	153.8 (15.3)	440.3 (90.7)				9.8 (0.7)	13.4 (2.7)	73	170 (11.3)	511.6 (45.2)				10.5 (0.6)	15.8 (1.4)
3.	Magnesius, 2016	20	111.0 (17.4)	451.5 (88.6)	0.26 (0.07)	10.3 (3.6)				29	119.1 (12.5)	535.1 (32.8)	0.22 (0.02)	13.1 (2.0)			
4.	Krismer, 2019	18						4.0 (0.4)	9.0 (1.7)	26						3.9 (0.3)	9.7 (0.8)
5.	Madetko, 2022	21	108 (19)	459 (65)	0.24 (0.05)	10.8 (2.5)	2.6 (0.9)			16	121 (23)	496 (73)	0.25 (0.03)	11.2 (1.8)	2.3 (0.6)		

Supplementary Table 4. Analytical data regarding the number of patients per study group, mean values and standard deviations of all available MRI markers in studies of corticobasal syndrome patients included in the meta-analysis.

Study number	Study ID	Corticobasal syndrome								Controls							
		N	midbrain area	pons area	M/P area	MRPI 1	MRPI 2	midbrain volume	pons volume	N	midbrain area	pons area	M/P area	MRPI 1	MRPI 2	midbrain volume	pons volume
1.	Groschel, 2006	18	128.7 (17.4)							22	138.1 (25.6)						
2.	Borroni, 2010	16			0.23 (0.03)					25			0.255 (0.03)				
3.	Jabbari, 2020	17						5.5 (0.5)	14.1 (1.7)	139	128 (20)	497 (49)		9.6 (2.0)			
4.	Madetko, 2022	19	81 (22)	444 (61)	0.18 (0.05)	15.4 (5.0)	4.2 (1.7)			16	121 (23)	496 (73)	0,25 (0.03)	11,2 (1.8)	2,3 (0.6)		
